# Barriers and Recommended Interventions to Prevent Melioidosis in Northeast Thailand: A Focus Group Study Using the Behaviour Change Wheel

**DOI:** 10.1371/journal.pntd.0004823

**Published:** 2016-07-29

**Authors:** Pornpan Suntornsut, Nittayasee Wongsuwan, Mayura Malasit, Rungreung Kitphati, Susan Michie, Sharon J. Peacock, Direk Limmathurotsakul

**Affiliations:** 1 Mahidol-Oxford Tropical Medicine Research Unit, Faculty of Tropical Medicine, Mahidol University, Bangkok, Thailand; 2 Department of Disease Control, Ministry of Public Health, Nonthaburi, Thailand; 3 Centre for Outcomes Research Effectiveness, Research Department of Clinical, Educational and Health Psychology, University College London, London, United Kingdom; 4 London School of Hygiene and Tropical Medicine, London, United Kingdom; 5 Department of Tropical Hygiene, Faculty of Tropical Medicine, Mahidol University, Bangkok, Thailand; 6 Nuffield Department of Medicine, University of Oxford, Oxford, United Kingdom; Johns Hopkins Bloomberg School of Public Health, UNITED STATES

## Abstract

**Background:**

Melioidosis, an often fatal infectious disease in Northeast Thailand, is caused by skin inoculation, inhalation or ingestion of the environmental bacterium, *Burkholderia pseudomallei*. The major underlying risk factor for melioidosis is diabetes mellitus. Recommendations for melioidosis prevention include using protective gear such as rubber boots and gloves when in direct contact with soil and environmental water, and consuming bottled or boiled water. Only a small proportion of people follow such recommendations.

**Methods:**

Nine focus group discussions were conducted to evaluate barriers to adopting recommended preventive behaviours. A total of 76 diabetic patients from northeast Thailand participated in focus group sessions. Barriers to adopting the recommended preventive behaviours and future intervention strategies were identified using two frameworks: the Theoretical Domains Framework and the Behaviour Change Wheel.

**Results:**

Barriers were identified in the following five domains: (i) knowledge, (ii) beliefs about consequences, (iii) intention and goals, (iv) environmental context and resources, and (v) social influence. Of 76 participants, 72 (95%) had never heard of melioidosis. Most participants saw no harm in not adopting recommended preventive behaviours, and perceived rubber boots and gloves to be hot and uncomfortable while working in muddy rice fields. Participants reported that they normally followed the behaviour of friends, family and their community, the majority of whom did not wear boots while working in rice fields and did not boil water before drinking. Eight intervention functions were identified as relevant for the intervention: (i) education, (ii) persuasion, (iii) incentivisation, (iv) coercion, (v) modeling, (vi) environmental restructuring, (vii) training, and (viii) enablement. Participants noted that input from role models in the form of physicians, diabetic clinics, friends and families, and from the government via mass media would be required for them to change their behaviours.

**Conclusion:**

There are numerous barriers to the adoption of behaviours recommended for melioidosis prevention. We recommend that a multifaceted intervention at community and government level is required to achieve the desired behaviour changes.

## Author Summary

Melioidosis is a serious infectious disease caused by the Gram-negative environmental saprophyte, *Burkholderia pseudomallei*. Infection in humans occurs following skin inoculation, inhalation or ingestion. Recommendations for melioidosis prevention include using protective gear such as rubber boots and gloves when in direct contact with soil and environmental water, and consuming bottled or boiled water. Northeast Thailand is a hot spot for melioidosis, but only a small proportion of people follow such recommendations. Here, we evaluated barriers to the adoption of preventive behaviours in diabetics (who are at highest risk for melioidosis), and systematically identified key functions required for future interventions. Our study participants had no knowledge of the disease, believed that there was no harm in not adopting the recommended preventive behaviours, and were not inclined to use boots and gloves while working in muddy rice fields. Participants reported that input from numerous role models (physicians, diabetic clinics, friends and families), and from the government via mass media would be required for them to change their behaviours. We recommend that a multifaceted intervention at community and government level is required to bring about the desired changes.

## Introduction

Melioidosis is a serious community-acquired infectious disease caused by the Gram-negative bacillus *Burkholderia pseudomallei*, which is present in soil and water in many tropical countries in Central and South America, sub-Saharan Africa, South Asia, Southeast Asia and northern Australia [1, 2]. The bacterium is intrinsically resistant to a wide range of antimicrobials, and treatment with ineffective antimicrobials has case fatality rates exceeding 70% [3]. An estimated 165,000 human melioidosis cases occur each year worldwide, of which 89,000 (54%) die [4]. In northeast Thailand, melioidosis is the second most common cause of community-acquired bacteremia, and the number of people dying there from melioidosis is now comparable to deaths from tuberculosis, and exceeds those from malaria, diarrheal illnesses and measles combined [5, 6]. Diabetes mellitus is the major underlying risk factor for melioidosis, and is present in more than 50% of all melioidosis cases [3]. The risk of people with diabetes acquiring melioidosis is about 12 times higher than the rest of the population [6, 7]. People with diabetes are therefore the major target population for melioidosis preventive measures [8].

Melioidosis is potentially preventable, as infection occurs by skin inoculation, inhalation or ingestion of bacteria in soil and water in endemic areas [3]. No melioidosis vaccine is currently available for human use [8]. Evidence-based guidelines for the prevention of melioidosis in Thailand recommends that residents and visitors should avoid direct contact with soil and water, wear protective gear such as boots and gloves when in direct contact with soil or water and only drink bottled or boiled water [9]. These recommendations have been repeatedly promoted by the Ministry of Public Health (MoPH) of Thailand to prevent leptospirosis, particularly since the sharp increase in the incidence of leptospirosis in Thailand between 1997 and 2000 [10]. In addition, in 2012 the MoPH launched a platform for improved preparedness, response and prevention to infectious diseases under a ‘One health concept’, which recognizes that human health is strongly connected to animal health and the environment [11]. Nonetheless, our previous study found that only a small proportion of people living in northeast Thailand followed such recommendations [9]. For example, many people still work in rice fields without protective gear, and drink untreated water [9]. In addition, there is no national campaign that is specific for melioidosis, public awareness of melioidosis is very low, and more than 90% of Thais have no knowledge of the disease [12].

Changing behaviour is typically complex, and a systematic approach is required to understand factors that influence adherence to recommendations so as to inform the design of future preventive interventions. In general, providing information alone does not change their behaviour [13, 14]. Frameworks have been developed that structure a wide range of possible influences on behaviour, including the Theoretical Domains Framework (TDF) and associated Behaviour Change Wheel (BCW [15–17]). The TDF is a useful framework for understanding the barriers and factors influencing specific behaviours [15, 16, 18, 19], while the BCW is a comprehensive framework that links this understanding to design interventions [17, 20]. Examples of using these frameworks to investigate implementation problems are the delivery of sepsis care bundles [21], antibiotic stewardship in healthcare facilities [22], and changing dietary behaviours in overweight children [23].

In this study, our aim was to evaluate barriers to and facilitators of behaviours recommended for melioidosis prevention. We then systematically identified key functions of interventions likely to be effective in increasing adherence to recommendations.

## Materials and Methods

### Focus group interviews

Focus group interviews were conducted to evaluate barriers to adopting (i) the use of protective gear such as rubber boots and gloves during direct contact with soil and environmental water, and (ii) the consumption of bottled or boiled water. These behaviours were selected from the list of recommendations for melioidos is prevention [9] on the assumption that these would be highly effective if adopted. The questions for the focus group interviews were grouped according to the Theoretical Domains Framework (TDF) (Table 1 and S1 Table), which comprises 14 key domains related to behaviour (S2 Table). TDF was chosen because it has been validated and proved useful to inform interventions aimed at bringing about behavioural changes [15].

**Table 1:** Schedule of questions for preventive behaviours for melioidosis*.

Domains **	Interview questions
Knowledge	Do you know about melioidosis
	If yes, what is your understanding of the disease?
	If no, description of the disease is explained to participant
	Do you know about preventive behaviours for melioidosis?
	If yes, what is your understanding of those behaviors?
	(Description of preventive behaviours for melioidosis is provided to participant)
Skills	Do you currently do your activities similar to recommended preventive behaviours for melioidosis?
	If yes, can you give an example?
	If no, can you explain the way you normally do your activities?
Social/Professional Role and Identity	What are your views about the preventive behaviours for melioidosis?
Beliefs about Capabilities	How difficult or easy is it for you to do preventive behaviours for melioidosis?
	What problems have you encountered?
	What would help you to overcome these problems?
Optimism	How confident are you that the preventive behaviours for melioidosis can prevent you from disease?
Beliefs about Consequences	What do you think the advantages are of doing preventive behaviours for melioidosis?
	What are the disadvantages?
	Would you say that the advantages outweigh the disadvantages?
Reinforcement	What type of support could influence you to do preventive behaviours for melioidosis?
	What type of media could influence you to do preventive behaviours for melioidosis?
	Do you have any suggestions on how to influence diabetics in general to do preventive behaviours for melioidosis?
Intention and goals	How much do you feel you should follow preventive behaviours for melioidosis?
	Are there time constraints to do preventive behaviours for melioidosis?
Memory, Attention and Decision Processes	Do you think you will do preventive behaviours for melioidosis?
	What are the reasons for deciding to do or not to do preventive behaviours for melioidosis?
Environmental Context and Resources	What do you think about resource or environment to do preventive behaviours for melioidosis?
	Do you have the necessary resources available for you to do preventive behaviours for melioidosis?
	What should healthcare facilities do for preventive behaviours for melioidosis?
	What should central government do for preventive behaviours for melioidosis?
Social Influences	Do you observe any of your families or neighbors doing preventive behaviours for melioidosis?
	What do you think about that?
	To what extent do your families or neighbors facilitate or hinder you to do preventive behaviours for melioidosis?
Emotion	Are there any other factors that influence you to do or not to do preventive behaviours for melioidosis?
Behavioral Regulation	Are there any ways that can help you to do preventive behaviours for melioidosis?

* Preventive behaviours for melioidosis include (i) using protective gear such as rubber boots and rubber gloves if direct contact with soil and environmental water is necessary and (ii) consuming bottled or boiled water. See S1 Table for the Thai translation of the interview questions.

** The questions were structured by using Theory Domain Framework (TDF [15]).

In April 2012, the focus group interviews were conducted at Det Udom Royal Crown Prince Hospital, Warin Chamrap hospital and Don Mot Daeng hospital, Ubon Ratchathani province, northeast Thailand, where melioidosis is highly endemic [6]. These three district hospitals were chosen based on the comparable size of the hospital (range from 30 to 90 beds) and distance from the center of Ubon Ratchathani province (within 100 kilometers). The majority of the population in these districts live in rural settings, and most adults (around 80%) are engaged in agriculture, particularly rice farming. Diabetic patients who came to follow-up visits at diabetic clinics were invited in sequential order to participate in focus group interviews. Eligible participants were males and females aged 18–60 years old who had been diagnosed with diabetes for at least 3 months. Diabetics in northeast Thailand were selected as the study population because they are at the highest risk of melioidosis and are the target population for prevention [24]. We excluded participants with a history of melioidosis. Participants were encouraged to compare and discuss their viewpoints within the group. Nittayasee Wongsuwan acted as a moderator and probed participants to elaborate on comments as necessary. After there were no new viewpoints raised, indicating saturation point, no further interviews were conducted. Each focus group lasted about 60–90 minutes.

### Data analysis

Data from focus group discussions were recorded in video formats, and detailed notes were taken by a note taker (Mayura Malasit). All videos were transcribed verbatim, and supplementary notes were added to ensure that all relevant participant comments and ideas were captured. To familiarize themselves with the data, two of the authors (Pornpan Suntornsut and Direk Limmathurotsakul) watched the videos and read the transcripts twice.

First, we used TDF and a deductive analytic process to classify responses [25, 26] according to the domains within the TDF [15]. For instances where coding differed between coders, differences of interpretation were discussed and an agreement was reached by consensus.

Second, we used a second framework, the Behaviour Change Wheel (BCW [17]) to identify the intervention functions most likely to be effective in changing the TDF domains identified. BCW was chosen because it was developed systematically, fitted well with the TDF [15], and has been found to be a robust starting point for designing interventions and planning policy [20]. Details of the BCW and explicit links between TDF domains and the BCW are given in the BCW guide [20]. In brief, the BCW is composed of a simple model of behaviour, COM-B, comprised of Capability, Opportunity, Motivation and Behaviour [20]. This sits at the hub of the ‘wheel’ linked to an inner ring of nine intervention functions (education, persuasion, incentivisation, coercion, training, restriction, environmental restructuring, modeling and enablement) which are in turn linked to an outer ring of seven policy categories (environment/social planning, communication/marketing, legislation, service provision, regulation, fiscal measures and guidelines [17, 20]). COM-B components represent sources of behaviour, which would need to be changed for the desired behaviour to occur. Capability is divided into physical and psychological, opportunity into physical and social, and motivation into reflective and automatic [17, 20]. We then linked general intervention functions to specific behaviour change techniques (BCTs) [27]. We used a set of criteria called APEASE to select the most appropriate intervention functions, BCTs and modes of delivery for our setting. APEASE criteria are (i) Affordability, (ii) Practicability, (iii) Effectiveness/cost-effectiveness, (iv) Acceptability, (v) Safety/side-effects and (vi) Equity in making context-based decisions [17, 20].

### Ethics

Approval for the study was obtained from the Ethics Committee of the Faculty of Tropical Medicine, Mahidol University, Bangkok, Thailand. Written informed consent was obtained from each participant prior to conducting each focus group interview. The funders had no role in study design, data collection and analysis, decision to publish, or preparation of the manuscript.

## Results

A total of 76 diabetic patients participated in nine focus group interviews (a median of 8 diabetic patients per group, ranging from 7 to 11). Overall, 20 (26%) were male and 56 (74%) were female. The median age of participants was 54 years (interquartile range, 47 to 61 years). Fifty-five participants (72%) were rice farmers and 13 (17%) were non-rice farmers. The majority of participants attained primary school education (89%; 68/72) and had a personal income less than 5,000 baht/month (equivalent to about 140 dollars/month; 86%; 64/72). Information about education and socioeconomic level was not available from two participants.

### Barriers influencing behaviours for melioidosis prevention

Most participants had no knowledge of the disease, believed that there was no harm in not adopting the recommended preventive behaviours, and were not inclined to use boots and gloves while working in muddy rice fields. Also, participants tended to drink water without boiling. Factors influencing the behaviours recommended for melioidosis prevention were related to five domains: (i) knowledge, (ii) beliefs about consequences, (iii) intention and goals, (iv) environmental context and resources; and (vi) social influences (Table 1). These were elaborations of four COM-B components: ‘psychological capability’, ‘reflective motivation’, ‘physical opportunity’ and ‘social opportunity’ (Table 2).

**Table 2:** Recommended intervention functions to achieve behaviour changes for melioidosis prevention in northeast Thailand.

Barrier domains *	Details of Barriers	COM-B components **	Recommended intervention functions *
Knowledge	Most participants have never heard of melioidosis, and none knew how to prevent the disease.	Psychological capability	Education
Beliefs about Consequences	Most participants thought that there was no harm in not doing the recommended preventive behaviours	Reflective motivation	Education, Persuasion and Modelling
Intentions and goals	Most participants had time constraints and had no intention to perform the recommended preventive behaviours	Reflective motivation	Education, Persuasion, Incentivisation, Coercion, and Modelling
Environmental Context and Resources	Rubber boots and rubber gloves were not practical for walking in the muddy rice fields while planting rice.	Physical opportunity	Environmental restructuring, Training, and Enablement
Social Influences	Participants normally followed what their friends and families did and recommended.	Social opportunity	Environmental restructuring and Enablement

* Barriers were identified by using focus group interviews and the Theory Domain Framework (TDF), and recommended intervention functions were identified by the Behaviour Change Wheel (BCW [17]) and APEASE criteria [20].

** COM-B component stands for Capability (Physical capability or Psychological capability), Opportunity (Physical opportunity or Social opportunity), Motivation (Automatic motivation or Reflective motivation)–Behaviour, represents source of the behaviours and is the core of the BCW [17].

#### Knowledge

We found that 72 of 76 participants (97%) had never heard of melioidosis, and many participants thought that melioidosis was a new disease. Four participants had heard of the disease. Two had heard from doctors because their relatives had died of melioidosis, and the other two from a public health volunteer (n = 1) and a friend (n = 1).

“(Is it) new disease? (I have) never heard of it”

(Male)

“This is the first time hearing of it.”

(Female)

“(I have) heard of it. My sister had liver abscess after working in rice field. Doctor told that she had melioidosis.”

(Female)

“I do not know much about it. My son had high fever and could not work. At first doctor told us that he had leptospirosis. He was transferred to the big hospital. He died. The doctor said he died of melioidosis infection in the blood”

(Female)

None of the participants knew how to prevent melioidosis, including the four who had heard of the disease.

#### Beliefs about consequences

After informing participants about behaviours recommended for melioidosis prevention, we found that participants reported no problems in terms of skills and beliefs about their capabilities to perform such behaviours. They reported that they could wear boots and boil water before drinking if they had to. Four participants stated that they always wore boots while working in rice fields to prevent leptospirosis (n = 2) and cuts from golden apple snails (n = 2). Twelve participants stated that they always boil water before drinking. However, most participants in every session thought that there was no harm in not adopting the recommended preventive behaviours. They considered that it was acceptable to work in rice fields without wearing boots. They believed that water from wells, boreholes, collected rainwater and tap water was clean and could be consumed without boiling.

“All of my friends and neighbors do not use boots, and none of them get sick”

(Male)

“I thought that there was no problem; so, I work in rice fields as usual (without boots)”

(Male)

“I drink rain water. I thought that it is clean. Rain water comes from the sky; so, I do not boil.”

(Female)

Participants felt that they would do the preventive behaviours recommended if they knew that the disease was fatal and that the advantages of the preventive behaviours were communicated to them.

“If there is an campaign, I will wear boots”

(Male)

“If it is recommended, I will buy and wear boots”

(Female)

#### Intention and goals

Lack of intention, reported as due to time constraints, were said to be barriers for the preventive behaviours recommended. Participants were not willing to work longer hours because working in muddy rice fields while wearing boots was slower than without boots. The time constraint was critical when they had to work quickly during the beginning of rice planting season. They also stated that boiling water took a considerable amount of time, including time to boil and time to wait for the water to cool down before drinking.

“My neighbors finished the work. I cannot even check whether I have wounds on my feet. I need to hurry up and finish the work (without wearing boots).”

(Male)

“No, I don’t boil (water) because (I am) tired after working and I would like to drink water immediately.”

(Male)

“It takes time for boiling. After boiling, it cannot be drunk immediately and we have to wait further”

(Male)

Some participants suggested boiling water in advance so that it was readily available, and boiling after cooking so that it could be done routinely and save resources.

“We can boil water, and save them in refrigerator.”

(Female)

“After cooking meal when the stove is still hot, we can boil water (efficiently).”

(Female)

#### Environmental context and resources

Lack of time as described above was one resource reported as a barrier.

**Fig 1:**
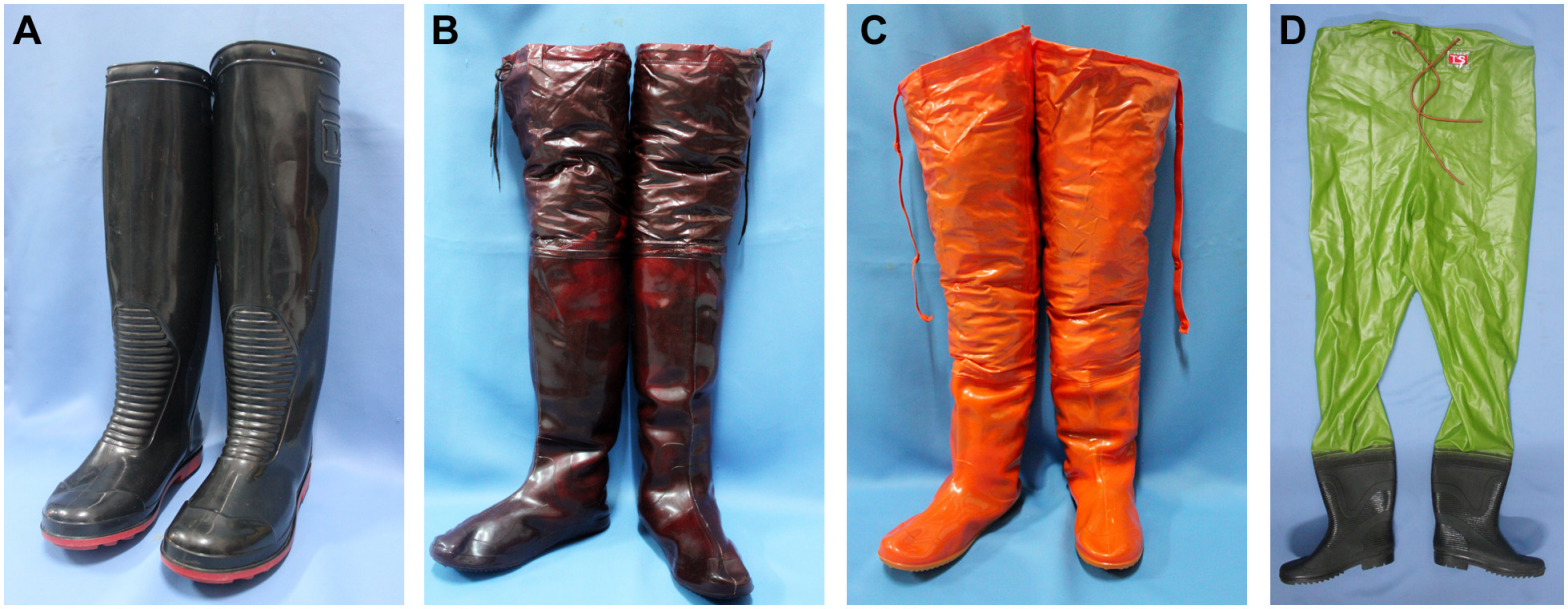
Wellington boots (a), over-the-knee boots (b), hip boots (c) and half-body waders (d).

A second very important contextual factor was the nature of the protective boots. Many participants pointed out that rubber boots and rubber gloves were not practical for walking in muddy rice fields while planting rice. Participants stated that it was too hot and humid to wear rubber boots while working all day under the sun. In addition, mud in flooded rice field sucked the rubber boots, making it very difficult to walk. All participants understood that the boots were Wellington boots (Fig 1), were made of rubber, and were below the knee-level and not fitted to the leg. This knowledge reflected the fact that Wellington boots were regularly given to rice farmers for free from government authorities.

“Mud sucks the boots. I can wear during harvesting rice (when rice fields are not flooded, and the soil are not muddy), but not during planting rice (when rice fields are flooded and the soil are muddy).”

(Male)

“It is too hot and humid to wear (boots). It is too heavy to walk.”

(Male)

“Rubber gloves do not fit. Now I use cloth gloves, cut the index finger and thumb (of the gloves) out, and use them during planting rice. We need to use thumb while planting rice.”

(Male).

When the moderator showed them over-the-knee boots, hip boots and half-body waders (Fig 1), which were commonly used in Bangkok during severe flood in 2012, many participants commented that those could solve the problem of walking difficulty in rice fields but did not solve the problem of heat and humidity. They considered that they could perform the recommended preventive behaviours more often or for longer hours if these boots were available. A participant stated that wearing long socks before wearing these boots could alleviate the problem of heat and humidity.

“(I have) never seen them”

(Female)

“These (boots) are light and seem wearable”

(Male)

“These are still hot and humid”

(Female)

“Wearing long socks together with these boots might be helpful”

(Female)

A third contextual factor was a lack of educators. The role of health care officers at diabetic clinics in the community hospitals was found to be important. Participants suggested that education about the disease and preventive behaviours should be provided at diabetic clinics.

“We need doctor to educate us”

(Male)

“(You) must announce (about this disease) to all diabetics in the (diabetic) clinic”

(Male)

“Sitting in a circle and talking like this is a good way to educate us in the (diabetic) clinics”,

(Female)

Participants also stated that the central government should promote melioidosis prevention through mass media such as television, community radio and news broadcasting tower. Participants noted that the disease and the benefit of preventive behaviours were of doubtful significance as they had never heard of these via the mass media.

“The government was not promoting this disease. I have heard of the influenza and leptospirosis but not this disease”

(Male)

“Many people hear things via broadcasting tower”

(Male)

“If the government do not tell, I will not know”

(Male)

“Why have I never heard anything (about this disease) in television?”

(Female)

#### Social influences

Participants reported that they normally followed what their friends, families and communities did and recommended. Social influence was raised as a very important factor in all sessions. Participants stated that they did not wear boots while working in rice fields and did not boil water before drinking, particularly because their friends, families and communities did not do so. Participants also recommended that their family members should be educated about the recommended preventive behaviours as they can influence behaviour.

“I normally follow what my friends do”

(Male)

“(You) need to give information to family members as well.”

(Female)

“Family members can stop (the behaviours at risk) and warn (them)”

(Female)

“Head of the community needed to lead this”

(Male)

“Community Health Volunteer needed to help us”

(Female)

### Recommended components of future interventions to support behaviour changes

Guided by the links between COM-B and intervention functions (S3 Table), we identified nine intervention functions that could be used to change the targeted COM-B components. We found that ‘restriction’ would not be applicable in our context. Therefore, the recommended intervention functions included ‘education’, ‘persuasion’, ‘incentivisation’, ‘coercion’, ‘modelling’, ‘environmental restructuring’, ‘training’ and ‘enablement’ (Table 2).

Guided by links between Intervention functions and BCTs (S4 Table), we considered that BCTs appropriate for our context included ‘information about health consequences’, ‘feedback on behaviour’, ‘feedback on outcomes of the behaviour’, ‘prompts/cues’, ‘self-monitoring of behaviour’, ‘credible source’, ‘demonstration of the behaviour’, ‘instruction on how to perform a behaviour’, ‘commitment’, ‘behavioural practice/rehearsal’, ‘adding objects to the environment’, ‘restructuring the physical environment’, ‘social support’, and ‘goal setting’. Examples of each BCT were developed and are listed in Table 3.

**Table 3:** Recommended behaviour change techniques (BCTs) to achieve behaviour changes for melioidosis prevention in northeast Thailand.

Recommended BCTs *	Intervention functions **	Examples of BCTs based on local context
Information about health consequences	Education and Persuasion	Explain that not wearing boots and gloves while working in rice fields and that drinking untreated water can lead to an often fatal infectious disease called melioidosis
Credible source	Persuasion	Present a speech given by a high status professional in the government to emphasise the importance of melioidosis prevention
		Ask doctors, nurses, head of villages, community health volunteers and the government to provide regular information about melioidosis and its prevention
Adding objects to the environment	Environmental restructuring and Enablement	Provide over-the-knee boots, hip boots or half-body waders
		Provide baby powder and long socks
Restructuring the physical environment	Environmental restructuring and Enablement	- Advise to apply baby powder and wear long socks before wearing boots
		Advise to place the kettle next to the stove so that it is convenient to boil water after cooking
		Advise to keep the boiled water in containers so that it is convenient to drink boiled water
Instruction on how to perform a behavior	Training	Show how to wear over-the-knee boots, hip boots and half-body waders
Demonstration of the behavior	Training and Modelling	Present video clips showing that wearing over-the-knee boots, hip boots and half-body waders can walk in the flooded or muddy rice fields without problems
Commitment	Incentivisation and Coercion	Ask the person to use an “I will” statement to affirm a strong commitment to start and continue wearing boots and gloves while working in rice fields and drinking only boiled water
Prompts/cues	Education and Environmental restructuring	Provide a large calendar with photos of the person wearing boots and gloves and drinking boiled water (by using an instant camera) to remind the person to wear boots and gloves while working in rice fields and to drink only boiled water
		Ask the person to place the boots and gloves next to the door
Self-monitoring of behavior	Education, Training, Enablement, Incentivisation and Coercion	Ask the person to record daily, in the calendar, whether they wear boots and gloves, and drink boiled water
Goal setting	Enablement	Set a goal as an outcome of changed behaviour (e.g. wearing boots 100% of the times working in rice fields and drinking only boiled waters)
Feedback on behavior	Education, Persuasion, Training, Incentivisation and Coercion	Ask the family of the person and community heath volunteers to observe and inform the person as to how often they wear boots and gloves while working in rice fields, and how often they drink boiled water
Feedback on outcome(s) of behavior	Education, Persuasion, Training, Incentivisation and Coercion	Inform the person of their final diagnosis and whether the person has melioidosis, if the person is admitted to hospital
Social support	Enablement	Give information about a group of community health volunteers that offer support for the behaviours
		Ask nurses, doctors, head of the villages, and families of the person to encourage continuation with the behaviours

* BCT is defined as an active component of an intervention designed to change behaviour [27]. Recommended BCTs were identified by the behaviour change wheel (BCW [17]) and APEASE criteria [20].

** A BCT may have more than one function[27].

Guided by links between Intervention functions and policy categories (S5 Table), we considered that the following policies would support the delivery of intervention functions for our context: ‘communication/marketing’, ‘guidelines’, ‘environmental/social planning’ and ‘service provision’. We found that ‘fiscal measures’, ‘regulation’ and ‘legislation’ would not be applicable in our context.

Using a taxonomy of modes of delivery for intervention functions (S1 Fig), we considered that the distance individual mode (phone helpline, mobile phone text and individually accessed computer programme) was unpractical and irrelevant to our setting. Therefore, the recommended modes of delivery included face-to-face mode (individual and group levels) and distance mode (population level). Distance mode at population levels could include broadcast media (television and radio), outdoor media (posters and billboards), digital media (internet and mobile phone applications) and print media (leaflets, newspaper and other written materials).

## Discussion

This study shows that the barriers for melioidosis prevention in Thailand are related to multiple domains, and we suggest that multiple intervention functions, BCTs and policies are required for the changes to be successful. The barriers identified are not limited to a lack of knowledge of the disease and measures to prevent it, and so providing information alone is unlikely to lead to the necessary behaviour changes. They also believe that there is no harm in not adopting the recommended preventive behaviours and give reasons for not boiling water due to lack of time and not using rubber boots and gloves in muddy rice fields due to discomfort. Understanding these barriers are also crucial as they point to the behaviours that require modification in order for the prevention to be effective. To our knowledge, this kind of systematic approach, using frameworks such as TDF and BCW to evaluate barriers and design an intervention after recommendations have been developed [10] is rarely performed in Thailand. This is highlighted by the fact that many people still work in rice fields without protective gear [9], despite the fact that wearing protective gear has been recommended in Thailand for many years [10, 11]. Our recommendations are based on the identified barriers, a systematic approach and local context. Furthermore, no single recommended BCTs or policy changes could affect all of the barriers. Therefore, all of the recommendations will need to be considered for the development of future interventions for melioidosis prevention in northeast Thailand.

Finding that most of our focus group participants (95%) have never heard of melioidosis is not surprising. Our result is comparable with the previous national survey finding that 74% of adults in Thailand have not heard of melioidosis [12]. The difference (95% vs. 74%) could be mainly because the survey respondents in the previous study were relatively younger and had higher education levels [12]. Lack of knowledge was found to be a major barrier in behaviour changes for many infectious diseases in Southeast Asia, including chronic obstructive pulmonary disease in Malaysia [28], sexual transmitted diseases in Cambodia [29] and liver fluke infection in Thailand [30]. Having accurate knowledge of the disease is fundamental for people to change their behaviour [17]. Therefore, providing knowledge about melioidosis and its prevention is still needed, and should be one of the main components in behaviour change interventions.

It was striking that most participants believed that there was no harm in not adopting the recommended preventive behaviours. Our finding is consistent with several studies in tropical countries where beliefs about consequences are one of the major barriers to the adoption of preventive behaviours. For example, Filipino farmers and laborers believed that wearing gloves during spraying pesticides would cause an illness called *pasma* rather than protecting themselves from pesticides [31]. Cambodian parents thought that HPV vaccine was unnecessary as they had traditional beliefs that whatever was going to happen would happen [32]. As we identified that ‘belief about consequences’ is a major barrier for melioidosis prevention, future interventions should also include ‘persuasion’ and ‘modeling’ as part of main intervention. This could be delivered through several BCTs in the interventions, as shown in Table 3.

A major concern raised by the focus group participants was that Wellington boots are hot and make walking difficult in muddy rice fields. Environmental context and resources might have been frequently overlooked in previous campaigns, in which Wellington boots have frequently been provided to farmers in Thailand as part of the previous campaign to prevent leptospirosis. The problem of boots was also raised in studies in other tropical developing countries in Sri Lanka [33] and Philippines [31]. Our pilot studies show that over-the-knee boots, hip boots and half-body waders can be used in flooded rice fields without causing difficulty in walking, but may still be uncomfortable in hot weather. Further studies are needed to focus on developing and trialing specifically designed boots that could allow farmers to walk easily in muddy paddy fields and comfortably in tropical developing countries.

Our study highlights the importance of ‘credible source’ and ‘social support’ as possible components of an intervention. This is consistent with other tropical developing countries, where these factors are very important in rural settings. For example, a study of oral poliovirus vaccines in Nigeria showed that having religious leaders, town announcers and health workers as primary sources of health information were strongly related to an individual’s probability of receiving the vaccine [34]. A study of acute respiratory infections in Bangladesh showed the importance of family and community people in decision-making [35]. Our participants even questioned whether the burden of melioidosis was real because they have never seen any information or campaign from the government via mass media. Our systematic approach also shows that multiple policy categories are required. Although guidelines (as one of the policy categories) for melioidosis prevention are now available [9], additional guidelines including all changes to service provision for policy makers is still needed. This suggests that commitment and action by the government are essential for the preventive interventions to be successful.

Our study has several strengths. First, we used a systematic approach (TDF and BCW) to evaluate barriers to behaviours recommended for melioidosis prevention, and we provide a range of recommendations for policy makers to use for behaviour change interventions in the future. If the interventions are designed without a systematic approach, it is common that a number of relevant intervention functions, BCTs and modes of delivery could be overlooked [20]. This systematic approach is useful to encourage intervention designers to be comprehensive in considering all options to intervene and then to systematically select those that are most promising for the context as shown in the previous successful examples [21–23]. Second, we selected only two behaviours that we consider would be highly effective for melioidosis prevention. This is consistent with the recommendation of behaviour change theory that the intervention should initially focus to just one or a few behaviours, and that building on small successes is more effective than intervening many behaviours simultaneously [20]. Third, the target behaviours in our study are also target behaviours recommended under the ‘One Health concept’ that could prevent other infectious diseases such as leptospirosis and acute diarrhea if adopted [11].

The major limitation of this study is that the identified barriers and recommended interventions to prevent melioidosis may not be equally relevant to all age and socioeconomic groups of the diabetic population in Thailand and beyond. It is possible that some barriers vary and that the intervention functions would need to be adjusted based on local context.

In conclusion, we recommend that health care providers together with policy makers should consider multifaceted interventions for melioidosis prevention. Health care providers should focus on delivering behaviour change interventions based on our recommended BCTs. Policy makers should focus on delivering disease education and implementing its preventive measures through healthcare providers and, particularly, through mass media.

## Supporting Information

S1 Table Schedule of questions for preventive behaviours for melioidosis (in Thai). (PDF)

S2 Table Theoretical Domains Framework definitions and theoretical constructs. (DOCX)

S3 Table Links between Theoretical Domains Framework, COM-B components (Capability, Opportunity, motivation and behaviour components) and intervention functions. (DOCX)

S4 Table Links between intervention functions and most frequently used Behaviour Change Techniques (BCTs). (DOCX)

S5 Table Links between policy categories and BCW intervention functions. (DOCX)

S1 Fig Taxonomy of modes of delivery for intervention functions that involve communication. (TIF)
