# Supplementary material for: Barriers and Recommended Interventions to Prevent Melioidosis in Northeast Thailand: A Focus Group Study Using the Behaviour Change Wheel
Source: PLoS Negl Trop Dis. 2016 Jul 29;10(7):e0004823. doi: 10.1371/journal.pntd.0004823 (PMC4966968; doi:10.1371/journal.pntd.0004823)
Supplement: S1 Table — (PDF) [file pntd.0004823.s001.pdf]

S1 Table. Schedule of questions for preventive behaviours for melioidosis (in Thai)

**Domains**	**Interview questions**
Knowledge	มีใครเคยได้ยินชื่อ โรคเมลิออยด์ หรือ โรคเมลิออยโดสิสบ้างคะ/ครับ<br>ถ้ามี คุณรู้จักโรคเมลิออยด์ว่าอย่างไรบ้างคะ/ครับ<br>ถ้าไม่มี นักวิจัยจะอธิบายเกี่ยวกับโรคเมลิออยด์ให้กับผู้เข้าร่วมการศึกษา<br>มีใครทราบเกี่ยวกับวิธีการป้องกันตัวเองจากโรคเมลิออยด์บ้างคะ/ครับ<br>ถ้ามี คุณรู้จักวิธีการป้องกันโรคเมลิออยด์ว่าอย่างไรบ้างคะ/ครับ<br>(นักวิจัยอธิบายเกี่ยวกับการป้องกันโรคเมลิออยด์ให้กับผู้เข้าร่วมการศึกษา)
Skills	มีใครในตอนนี้ที่เวลาทำกิจวัตรต่างๆ ได้ทำตามที่แนะนำในการป้องกันโรคเมลิออยด์บ้างคะ/ครับ<br>ถ้ามี ช่วยยกตัวอย่างหน่อยคะ/ครับ<br>ถ้าไม่มี ช่วยเล่าให้ฟังหน่อยคะ/ครับ ว่าตอนนี้ทำกิจวัตรต่างๆ อย่างไร
Social/Professional Role and Identity	ในความคิดของแต่ละคน คิดอย่างไรกับวิธีการป้องกันโรคเมลิออยด์บ้างคะ/ครับ
Beliefs about Capabilities	คิดว่าการทำตามวิธีป้องกันโรคเมลิออยด์มีความยากหรือง่ายอย่างไรบ้างคะ/ครับ<br>คิดว่ามีปัญหาอะไรบ้างคะ/ครับ<br>คิดว่าอะไรจะช่วยให้สามารถขจัดปัญหาเหล่านั้นได้บ้างคะ/ครับ
Optimism	มีความมั่นใจอย่างไรในการที่จะทำตามที่แนะนำในการป้องกันโรคเมลิออยด์บ้างคะ/ครับ
Beliefs about Consequences	คิดว่าการทำตามคำแนะนำในการป้องกันโรคเมลิออยด์ มีประโยชน์อะไรบ้างคะ/ครับ<br>คิดว่าการทำตามคำแนะนำในการป้องกันโรคเมลิออยด์ มีโทษหรือทำให้เกิดผลเสียอะไรบ้างหรือเปล่าคะ/ครับ<br>คิดว่าการทำตามคำแนะนำในการป้องกันโรคเมลิออยด์ มีประโยชน์มากกว่าโทษหรือเปล่าคะ/ครับ
Reinforcement	คิดว่าต้องการการช่วยเหลือหรือการสนับสนุนอะไร ที่จะทำให้เรา (คุณ) ทำตามคำแนะนำในการป้องกันโรคเมลิออยด์บ้างคะ/ครับ<br>คิดว่าสื่อแบบไหน ที่จะทำให้เรา (คุณ) ทำตามคำแนะนำในการป้องกันโรคเมลิออยด์บ้างคะ/ครับ<br>มีใครมีข้อแนะนำในการที่จะทำให้เรา (คุณ) ทำตามคำแนะนำในการป้องกันโรคเมลิออยด์บ้างคะ/ครับ
Intention and goals	แต่ละคนคิดว่า จะทำตามคำแนะนำในการป้องกันโรคเมลิออยด์กันได้แค่ไหนบ้างคะ/ครับ<br>มีใครมีปัญหาเกี่ยวกับเรื่องเวลาในการทำตามคำแนะนำในการป้องกันโรคเมลิออยด์บ้างไหมคะ/ครับ
Memory, Attention and Decision Processes	แต่ละคนคิดจะทำตามคำแนะนำในการป้องกันโรคเมลิออยด์ไหมคะ/ครับ<br>อะไรเป็นสาเหตุให้ตัดสินใจว่าจะทำหรือไม่ทำตามคำแนะนำในการป้องกันโรคเมลิออยด์บ้างคะ/ครับ
Environmental	แต่ละคนคิดอย่างไร ของที่มี หรือสิ่งแวดล้อมต่างๆ มีส่วนเกี่ยวข้องกับการทำตามคำแนะนำในการป้องกันโรคเมลิ

Context and Resources	ออยด์บ้างคะ/ครับ<br>แต่ละคนมี ของใช้ที่จำเป็นในการทำตามคำแนะนำในการป้องกันโรคเมลิออยด์หรือเปล่าคะ/ครับ<br>โรงพยาบาลหรืออนามัยควรทำอะไรบ้าง เพื่อการป้องกันโรคเมลิออยด์บ้างคะ/ครับ<br>รัฐบาลควรทำอะไรบ้าง เพื่อการป้องกันโรคเมลิออยด์บ้างคะ/ครับ
Social Influences	แต่ละคน มีใครเคยเห็นคนในครอบครัว หรือเพื่อนบ้างทำตามคำแนะนำในการป้องกันโรคเมลิออยด์หรือเปล่าคะ/ครับ<br>แต่ละคนคิดว่าอย่างไรบ้างคะ/ครับ<br>แต่ละคนคิดว่าคนในครอบครัว หรือเพื่อนบ้าง มีส่วนช่วย หรือมีส่วนไม่ให้เรา (คุณ) ทำตามคำแนะนำในการป้องกันโรคเมลิออยด์หรือเปล่าคะ/ครับ
Emotion	แต่ละคนคิดว่า มีอะไรอย่างอื่น ที่ทำให้เราทำหรือไม่ทำตามคำแนะนำในการป้องกันโรคเมลิออยด์บ้างหรือเปล่าคะ/ครับ
Behavioral Regulation	แต่ละคนคิดว่า มีอะไรที่จะช่วยทำให้เราทำทำตามคำแนะนำในการป้องกันโรคเมลิออยด์บ้างหรือเปล่าคะ/ครับ
