# Supplementary material for: Barriers and Recommended Interventions to Prevent Melioidosis in Northeast Thailand: A Focus Group Study Using the Behaviour Change Wheel
Source: PLoS Negl Trop Dis. 2016 Jul 29;10(7):e0004823. doi: 10.1371/journal.pntd.0004823 (PMC4966968; doi:10.1371/journal.pntd.0004823)
Supplement: S2 Table — (DOCX) [file pntd.0004823.s002.docx]

**S2 Table.** Theoretical Domains Framework definitions and theoretical constructs

| **Domains (Definition)** | **Theoretical constructs** |
| --- | --- |
| **Knowledge**  (An awareness of the existence of something) | Knowledge (including knowledge of condition/scientific rationale)  Procedural knowledge  Knowledge of task environment |
| **Skills**  (An ability or proficiency acquired through practice) | Skills  Skills development  Competence  Ability  Interpersonal skills  Practice  Self-assessment |
| **Memory, attention and decision processes**  (The ability to retain information, focus selectively on aspects of the environment and choose between two or more alternative) | Memory  Attention  Attention control  Decision making  Cognitive overload/ tiredness |
| **Behavioral regulation**  (Anything aimed at managing or changing objectively observed or measured actions) | Self-monitoring  Breaking habit  Action plan |
| **Social/professional role and identity**  (A coherent set of behaviors and displayed personal qualities of an individual in a social or work setting) | Professional identity  Professional role  Social identity  Identity  Professional boundaries  Professional confidence  Group identity  Leadership  Organizational commitment |
| **Beliefs about capabilities**  (Acceptance of the truth, or validity about an ability, talent, or facility that a person can put to constructive use) | Self-confidence  Perceived competence  Self-efficacy  Perceived behavioral control  Beliefs  Self-esteem  Empowerment  Professional confidence |
| **Optimism**  (The confidence that things will happen for the best or that desired goals will be attained) | Optimism  Pessimism  Unrealistic optimism  Identity |
| **Beliefs about consequences**  (Acceptance of the truth reality, or validity about outcomes of a behavior in a given situation) | Beliefs  Outcome expectancies  Characteristics of outcome expectancies  Anticipated regret  Consequents |
| **Intentions**  (A conscious decision to perform a behavior or a resolve to act in a certain way) | Stability of intentions  Stages of change model  Transtheoretical model and stages of change |
| **Goals**  (Mental representations of outcomes or end states that an individual wants to achieve) | Goals (distal/proximal)  Goal priority  Goal/target setting  Goal (autonomous/controlled)  Action planning  Implementation intention |
| **Reinforcement**  (Increasing the probability of a response by arranging a dependent relationship or contingency, between the response and a given stimulus) | Rewards (proximal/ distal valued/ not valued, probable/ improbable)  Incentives  Punishment  Consequents  Reinforcement  Contingencies sanctions |
| **Emotion**  (A complex reaction pattern, involving experiential, behavioral, and physiological elements, by which the individual attempts to deal with a personally significant matter or event) | Fear  Anxiety  Affect  Stress  Depression  Positive/negative affect  Burn-out |
| **Environmental context and resources**  (Any circumstance of a person’s situation or environment that discourages or encourages the development of skills and abilities, independence, social competence, and adaptive behavior) | Environmental stressors  Resource/ martial resources  Organizational culture/ climate  Silent event/ critical incidents  Person x environment interaction  Barriers and facilitators. |
| **Social influences**  (Those interpersonal process that can cause individuals to change their thoughts. Feelings or behavior) | Social norms  Group conformity  Social comparisons  Group norms  Social supports  Power  Intergroup conflict  Alienation group identity  Modelling |
