# Supplementary material for: Barriers and Recommended Interventions to Prevent Melioidosis in Northeast Thailand: A Focus Group Study Using the Behaviour Change Wheel
Source: PLoS Negl Trop Dis. 2016 Jul 29;10(7):e0004823. doi: 10.1371/journal.pntd.0004823 (PMC4966968; doi:10.1371/journal.pntd.0004823)
Supplement: S3 Table — (DOCX) [file pntd.0004823.s003.docx]

**S3 Table.** Links between Theoretical Domains Framework, COM-B components (Capability, Opportunity, motivation and behaviour components) and intervention functions

| **Domains** | **COM-B components** | **Intervention functions** |
| --- | --- | --- |
| **Skills** | Physical capability | Training |
| **Knowledge** | Psychological capability | Education |
| **Memory, attention and decision processes** | Psychological capability | Training  Environmental restructuring Restriction  Enablement |
| **Behavioural regulation** | Psychological capability | Education  Training  Modelling  Enablement |
| **Environmental Context and Resources** | Physical opportunity | Environmental restructuring Restriction  Training  Enablement |
| **Social Influences** | Social opportunity | Environmental restructuring Restriction  Training  Enablement |
| **Professional/Social Role and Identity** | Reflective motivation | Education  Persuasion  Modelling |
| **Beliefs about Consequences** | Reflective motivation | Education  Persuasion  Modelling  Enablement |
| **Intentions** | Reflective motivation | Education  Persuasion  Incentivisation  Coercion  Modelling |
| **Goals** | Reflective motivation | Education  Persuasion  Incentivisation  Coercion  Modelling |
| **Reinforcement** | Automatic motivation | Training  Incentivisation  Coercion  Environmental restructuring |
| **Emotion** | Automatic motivation | Persuasion  Incentivisation  Coercion  Modelling  Enablement |
