# Supplementary material for: Barriers and Recommended Interventions to Prevent Melioidosis in Northeast Thailand: A Focus Group Study Using the Behaviour Change Wheel
Source: PLoS Negl Trop Dis. 2016 Jul 29;10(7):e0004823. doi: 10.1371/journal.pntd.0004823 (PMC4966968; doi:10.1371/journal.pntd.0004823)
Supplement: S4 Table — (DOCX) [file pntd.0004823.s004.docx]

**S4 Table.** Links between intervention functions and most frequently used Behaviour Change Techniques (BCTs)

| **Intervention functions** | **Frequently used BCTs** |
| --- | --- |
| **Education** | Information about social and environmental consequences  Information about health consequences  Feedback on behavior  Feedback on outcome(s) of behavior  Prompts/cues  Self-monitoring of behavior |
| **Persuasion** | Credible source  Information about social and environmental consequences  Information about health consequences  Feedback on behavior  Feedback on outcome(s) of behavior |
| **Incentivisation** | Feedback on behavior  Feedback on outcome(s) of behavior  Monitoring of behavior by others without evidence of feedback  Monitoring outcome of behavior by others without evidence of feedback  Self-monitoring of behavior |
| **Coercion** | Feedback on behavior  Feedback on outcome(s) of behavior  Monitoring of behavior by others without evidence of feedback  Monitoring outcome of behavior by others without evidence of feedback  Self-monitoring of behavior |
| **Training** | Demonstration of the behavior  Instruction on how to perform a behavior  Feedback on behavior  Feedback on outcome(s) of behavior  Self-monitoring of behavior  Behavior practice/rehearsal |
| **Environmental restructuring** | Adding objects to the environment  Prompts/cues  Restructuring the physical environment |
| **Modelling** | Demonstration of the behavior |
| **Enablement** | Social support (unspecified)  Social support (practical)  Goal setting (behavior)  Goal setting (outcome)  Adding objects to the environment  Problem solving  Action planning  Self-monitoring of behavior  Restructuring the physical environment  Review behavior goal(s)  Review outcome goal(s) |
