# Supplementary material for: Barriers and Recommended Interventions to Prevent Melioidosis in Northeast Thailand: A Focus Group Study Using the Behaviour Change Wheel
Source: PLoS Negl Trop Dis. 2016 Jul 29;10(7):e0004823. doi: 10.1371/journal.pntd.0004823 (PMC4966968; doi:10.1371/journal.pntd.0004823)
Supplement: S5 Table — (DOCX) [file pntd.0004823.s005.docx]

**S5 Table. Links between policy categories and BCW intervention functions**

| **Policy categories** | **Definition** | **Intervention functions** |
| --- | --- | --- |
| **Communication/marketing** | Using print, electronic, telephonic or broadcast media | Education  Persuasion  Incentivisation  Coercion |
| **Guidelines** | Creating documents that recommend or mandate practice. This includes all change to service provision | Education  Persuasion  Incentivisation  Coercion  Training  Restriction  Environmental restructuring Modelling  Enablement |
| **Fiscal measures** | Using the tax system to reduce or increase the financial cost | Incentivisation  Coercion  Training  Enablement  Environmental restructuring |
| **Regulation** | Establishing rules or principles of behaviour or practice | Education  Persuasion  Incentivisation  Coercion  Training  Restriction  Environmental restructuring Enablement |
| **Legislation** | Marking or changing laws | Education  Persuasion  Incentivisation  Coercion  Training  Restriction  Environmental restructuring Enablement |
| **Environmental/social planning** | Designing and/ or controlling the physical or social environment | Environmental restructuring Modelling |
| **Service provision** | Delivering a service | Education  Persuasion  Incentivisation  Coercion  Training  Modelling  Enablement |
