# Supplementary figures and images for: Barriers and Recommended Interventions to Prevent Melioidosis in Northeast Thailand: A Focus Group Study Using the Behaviour Change Wheel
Source: PLoS Negl Trop Dis. 2016 Jul 29;10(7):e0004823. doi: 10.1371/journal.pntd.0004823 (PMC4966968; doi:10.1371/journal.pntd.0004823)

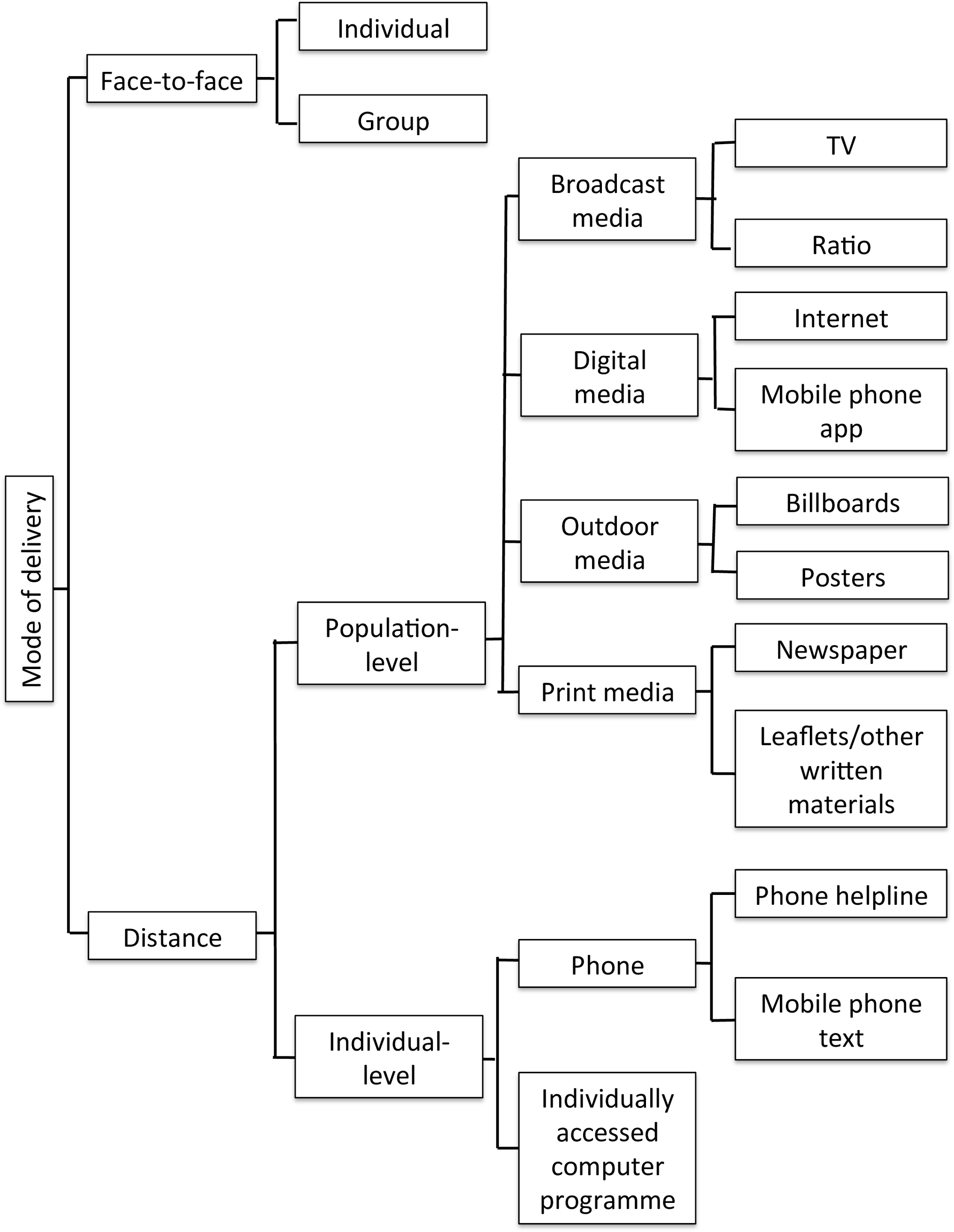

Supplement: S1 Fig — (TIF) [file pntd.0004823.s006.tif]
